# Quantitative Proteomics Reveal That Metabolic Improvement Contributes to the Cardioprotective Effect of T_89_ on Isoproterenol-Induced Cardiac Injury

**DOI:** 10.3389/fphys.2021.653349

**Published:** 2021-06-28

**Authors:** Xiao-Hong Wei, Xiao Guo, Chun-Shui Pan, Huan Li, Yuan-Chen Cui, Li Yan, Jing-Yu Fan, Jing-Na Deng, Bai-He Hu, Xin Chang, Shu-Ya He, Lu-Lu Yan, Kai Sun, Chuan-She Wang, Jing-Yan Han

**Affiliations:** ^1^Department of Integration of Chinese and Western Medicine, School of Basic Medical Sciences, Peking University, Beijing, China; ^2^Tasly Microcirculation Research Center, Peking University Health Science Center, Beijing, China; ^3^Academy of Integration of Chinese and Western Medicine, Peking University Health Science Center, Beijing, China; ^4^Key Laboratory of Microcirculation, State Administration of Traditional Chinese Medicine, Beijing, China; ^5^Key Laboratory of Stasis and Phlegm, State Administration of Traditional Chinese Medicine, Beijing, China; ^6^State Key Laboratory of Core Technology in Innovative Chinese Medicine, Tianjin, China

**Keywords:** glycolysis, fatty acid oxidation, energy metabolism, mitochondrial electron transport chain, cardiac hypertrophy

## Abstract

**Background:**

T_89_, a traditional Chinese medicine, has passed phase II, and is undergoing phase III clinical trials for treatment of ischemic cardiovascular disease by the US FDA. However, the role of T_89_ on isoproterenol (ISO)-induced cardiac injury is unknown. The present study aimed to explore the effect and underlying mechanism of T_89_ on ISO-induced cardiac injury.

**Methods:**

Male Sprague-Dawley rats received subcutaneous injection of ISO saline solution at 24 h intervals for the first 3 days and then at 48 h intervals for the next 12 days. T_89_ at dose of 111.6 and 167.4 mg/kg was administrated by gavage for 15 consecutive days. Rat survival rate, cardiac function evaluation, morphological observation, quantitative proteomics, and Western blotting analysis were performed.

**Results:**

T_89_ obviously improved ISO-induced low survival rate, attenuated ISO-evoked cardiac injury, as evidenced by myocardial blood flow, heart function, and morphology. Quantitative proteomics revealed that the cardioprotective effect of T_89_ relied on the regulation of metabolic pathways, including glycolipid metabolism and energy metabolism. T_89_ inhibited the enhancement of glycolysis, promoted fatty acid oxidation, and restored mitochondrial oxidative phosphorylation by regulating Eno1, Mcee, Bdh1, Ces1c, Apoc2, Decr1, Acaa2, Cbr4, ND2, Cox 6a, Cox17, ATP5g, and ATP5j, thus alleviated oxidative stress and energy metabolism disorder and ameliorated cardiac injury after ISO. The present study also verified that T_89_ significantly restrained ISO-induced increase of HSP70/HSP40 and suppressed the phosphorylation of ERK, further restored the expression of CX43, confirming the protective role of T_89_ in cardiac hypertrophy. Proteomics data are available *via* ProteomeXchange with identifier PXD024641.

**Conclusion:**

T_89_ reduced mortality and improves outcome in the model of ISO-induced cardiac injury and the cardioprotective role of T_89_ is correlated with the regulation of glycolipid metabolism, recovery of mitochondrial function, and improvement of myocardial energy.

## Introduction

Cardiovascular events due to ischemic heart disease contribute to the majority of deaths among cardiovascular disorders worldwide (Katz and Gavin, 2019). It is reported that patients with ischemic angina symptom but without significant coronary artery occlusion are more prone to sudden death, which is a high-risk factor to cardiovascular events (Jespersen et al., 2012). Isoproterenol (ISO) is a synthetic, non-selective β-agonist, which depletes the energy reserve of cardiomyocytes and induces myocardial ischemia and infarct-like necrosis, resulting in cardiac hypertrophy, remodeling, and heart failure (Karam et al., 2020).

It has been well evidenced that ISO-induced cardiac injury implicates metabolism disorders, involving abnormalities in glycolipid metabolism and energy metabolism (Stapel et al., 2017). It has been revealed that ISO could lead to less fatty acid entering in mitochondrion, resulting in suppression of fatty acid oxidation, while enhancing glycolysis by regulating glycolipid metabolism enzymes (Guo et al., 2016). ISO has also been demonstrated to induce mitochondrial oxidative phosphorylation impairment (Krestinina et al., 2020), which in turn, along with glycolipid disorders, resulted in reduction of ATP production. Mitochondrial electron transport chain (ETC) consists of transmembrane protein complexes (complex I to complex IV) and the freely moving electron transfer carrier-ubiquinone and cytochrome *c*, together with complex V (ATP synthase), which is the basis for ATP production during mitochondrial oxidative phosphorylation (Guo et al., 2018). Electrons produced by the oxidation of NADH or succinate by Complex I or II, respectively, are transferred to complex III *via* the pool of ubiquinone and then to complex IV (cytochrome *c* oxidase) *via* soluble cytochrome *c* (Guo et al., 2017). During the electron transferring from complex I to complex IV, an electrochemical gradient of proton is formed across the mitochondrial inner membrane, which drives complex V to catalyze ADP to produce ATP (Cadenas, 2018). Each ETC complex consists of several subunits, downregulation of which will lead to corresponding complex activity being restrained and ATP generation reduction (Letts et al., 2016; Signes and Fernandez-Vizarra, 2018).

T_89_ [also known as cardiotonic pills (CP), compound Danshen dripping pills in Chinese] is a widely used traditional Chinese medicine in China for treating ischemic angina pectoris, which consists of salvia miltiorrhiza (SM), panax notoginseng (PN), and Borneol. T_89_ has passed phase II and is undergoing phase III clinical trials for prevention and treatment of ischemic cardiovascular disease by the United States Food and Drug Administration since 2016 (Aa et al., 2019). Guo et al. (2016) observed that T_89_ pretreatment increased energy production and regulated the dominant energy metabolism mode accomplished with enhancing a metabolic shift toward fatty acid metabolism from glycolysis induced by ISO, which indicated that the regulation of energy metabolism was involved in the underling mechanism whereby T_89_ exerted effect on myocardial ischemia. In this work, the results were obtained by detecting the metabolite indices in plasma and heart tissue using metabolomics study, while there was little data regarding the molecular mechanism underlying T_89_ on ISO-induced cardiac injury. Our previous study has confirmed 3,4-dihydroxyl-phenyl lactic acid (DLA, a major ingredient of SM, also called Danshensu in Chinese) and total salvianolic acid could prevent I/R-induced myocardial injury *via* restoring mitochondrial ETC function (Yang et al., 2015; Huang R. et al., 2019), while ginsenoside Rg1 (Rg1) and ginsenoside Rb1 (Rb1), the main ingredient of PN, could protect against I/R-induced energy metabolism disorder by upregulating ATP 5D expression (Cui et al., 2017; Li et al., 2018). However, the cardioprotective effect and the mechanism for the effect of T_89_ on ISO-induced cardiac injury have not been fully understood. Therefore, the present study was designed to investigate the effect of T_89_ on cardiac injury after ISO challenge and to explore the underlying mechanism based on quantitative proteomic analyses.

## Materials and Methods

### Animals

Male Sprague-Dawley (SD) rats weighing 180–200 g were obtained from the Peking University Health Science Center Animal Center (Beijing, certificate no. SCXK 2006-0008) and handled according to the guidelines of the Peking University Health Science Center Animal Research Committee. All experimental procedures were approved by Peking University Biomedical Ethics Committee Experimental Animal Ethics Branch, complying with the “Guidelines for the Care and Use of Laboratory Animals”, published by the National Institutes of Health (Zheng et al., 2019).

### Reagents

T_89_ (270 mg per capsule, lot number: 20160601S) was obtained from Tasly Pharmaceutical Co., Ltd. (Tianjin, China), which was prepared from water-ethanol extract of SM, PN, and borneol under the guideline of Good Manufacturing Practice and Good Laboratory Practice verified by the Chinese, and United States Government agencies. The doses of T_89_ for rats were 111.6 and 167.4 mg/kg, equivalent to low and high dose for human in FDA phases II and III clinical trials. ISO was purchased from Tokyo Chemical Industry Co., Ltd. (product number: I0260).

### Animal Model and Treatments

The SD rats were randomly divided into five groups: (1) Sham group (Sham); (2) Treatment with 167.4 mg/kg T_89_ in Sham group (Sham + T_89_ 167.4); (3) ISO-induced model (ISO); (4) Treatment with 111.6 mg/kg T_89_ in ISO-induced model (ISO + T_89_ 111.6); and (5) Treatment with 167.4 mg/kg T_89_ in ISO-induced model (ISO + T_89_ 167.4). The rats in the Sham and ISO-induced groups received subcutaneous injections of normal saline and ISO saline solution (10 mg/kg), respectively, at 24 h intervals for the inchoate three consecutive days and then at 48 h intervals for the later 4–15 days. Rats treated with T_89_ in the ISO-induced groups were given corresponding drugs by intragastric administration for 15 consecutive days starting from 5 min before ISO injection. The animals in the Sham + T_89_ 167.4 group was given T_89_ at 167.4 mg/kg by intragastric administration for 15 consecutive days, while those in the Sham group received normal saline instead the same way.

### Rat Survival Rate

During the 15-day observation, survival of each group was monitored daily for 15 days. At the end of the experiment, survival curves were plotted and rats were anesthetized with 2% pentobarbital (60 mg/kg) by peritoneal injection.

### Myocardial Blood Flow

Two hours after ISO injection on Day 15 of the experiment, myocardial blood flow (MBF) in each group was measured using Laser-Doppler Perfusion Imager (PeriScan PIM3 System; PERIMED, Stockholm, Sweden) equipped with a computer. Briefly, heart was exposed after left thoracotomy, and a computer-controlled optical scanner directed a low-powered He–Ne laser beam over the exposed heart. The scanner head was positioned in parallel to the surface of the heart at a distance of 18 cm, and the beam illuminated the tissue to a depth of 0.5 mm. A color-coded image to denote specific relative perfusion level was displayed on a video monitor, and all images were evaluated with the software LDPIwin 3.1 (PeriScan PIM3 System; PERIMED, Stockholm, Sweden). The MBF magnitude is represented by different colors, with blue to red denoting low to high (Wei et al., 2013).

### Heart Function Test

Heart function was tested by a biofunction experiment system BL-420F (Chengdu Taimen Technology Ltd., Chengdu, China), which was connected to a cannulation inserted into the left ventricle through the right carotid artery. Heart rate (HR), left ventricular systolic pressure (LVSP), left ventricular diastolic pressure (LVDP), left ventricular end diastolic pressure (LVEDP), left ventricular maximum upstroke velocity (+dp/dt max), and left ventricular maximum descent velocity (-dp/dt max) were evaluated 2 h after ISO injection on the Day 15 of the experiment (Zheng et al., 2019).

### The Ratio of Heart Weight to Body Weight

On Day 15 of the experiment, 2 h after ISO injection, hearts were removed and washed with normal saline. Both heart weight and body weight were weighed, and heart weight to body weight (HW/BW) was calculated to evaluate the cardiac hypertrophy response to the procedure (Huang R. et al., 2019).

### Myocardial Histology and Immunofluorescence Staining

At the end of the experiment, rat hearts were removed and fixed in 4% paraformaldehyde. Serial paraffin sections (5 μm thickness) were prepared and stained with hematoxylin eosin (HE) as routine (Yan et al., 2018).

The immunofluorescence staining of wheat germ agglutinin (WGA, Invitrogen) and Connexin 43 (CX 43) was performed. Images of immunofluorescence were observed at ×40 magnification of objective with a Laser Scanning Confocal Microscope (TCS SP5, Leica, Mannheim, Germany). Cardiomyocyte cross-section area was determined on sections stained with WGA in randomly selected five fields, calculating the average of cross-section areas of 10 cells in each, using Image-Pro Plus 6.0 (Media Cybernetic, Bethesda, MD, United States) (Huang R. et al., 2019).

### Ultrastructure Examination

An approximately 1 mm^3^ fresh myocardial tissue block was taken from the left ventricle. Tissues were fixed with 3% glutaraldehyde and postfixed with 1% osmium tetraoxide. The specimens were processed as routine for ultrathin sections. Sections were stained with uranium acetate and lead citrate, and ultrastructural changes were then evaluated by using a transmission electron microscope (JEM-1230; JEOL, Tokyo, Japan) (Wei et al., 2013).

### Enzyme-Linked Immunosorbent Assay

Enzyme-linked immunosorbent assay (ELISA) was undertaken using a specific kit indicated by a microplate reader (Multiskan MK3, Thermo, New Bedford, MA, United States), respectively, to determine the content of creatine kinase MB (CK MB), and Troponin I (cTn I) in plasma (Huanya Biomedicine Technology Co., Ltd., Beijing, China); pyruvic acid, 3-hydroxybutyric acid (3-HYA), atrial natriuretic peptide (ANP), thiobarbituric acid reacting substances (TBARS), ATP, and AMP in the left ventricle (Nanjing Jiancheng Bioengineering Institute, Nanjing, China). Likewise, the activities of mitochondrial ETC complex I, IV, and V were also detected by ELISA kit according to the manufacturer’s instructions (Abcam, Cambridge, MA, United States) (Huang D. D. et al., 2019).

### Proteomics Analysis

At the end of 15-day observation, left ventricular tissue proteins from three groups (Sham, ISO, ISO + T_89_ 167.4) were extracted and quantified using the BCA Protein Assay Kit (Thermo Fisher Scientific, United States). The proteins were then labeled with tandem mass tags (TMT). TMT6/10 (Pierce, Rockford, IL, United States) with different reporter ions (126–131 Da) were applied as isobaric tags for relative quantification. Then the labeled peptide aliquots were combined for fractionation and further LC-MS analysis. The LC-MS/MS analysis was carried out in CapitalBio Technology by using Q Exactive mass spectrometer (Thermo Fisher Scientific, United States). Twenty most intense precursor ions from a survey scan were selected for MS/MS detected at a mass resolution of 35,000 at *m*/*z* of 400 in Orbitrap analyzer. All the tandem mass spectra were produced by higher-energy collision dissociation (HCD) method. The MS/MS data was collected and searched against the Oryctolagus cuniculus database using the SEQUEST algorithms. A protein with fold change ≥1.5, and the presence of least two unique peptides with a *P*-value < 0.05, was considered to be a differentially expressed protein (DEP). Finally, DEPs were analyzed by reference to three key databases: Gene Ontology (GO), Kyoto Encyclopedia of Genes and Genomes (KEGG), and Reactome and the Clusters of Orthologous Groups (COG) (Park et al., 2019). The mass spectrometry proteomics data have been deposited to the ProteomeXchange Consortium *via* the PRIDE (Perez-Riverol et al., 2019) partner repository with the dataset identifier PXD024641.

### Western Blotting Assay

Western blotting assay was performed to verify the differentially expressed proteins obtained from quantitative proteomics analysis. Myocardial tissues were taken from left ventricle, and total proteins were extracted using a protein extraction kit (Applygen Technologies, Beijing, China) and mixed with 5× electrophoresis sample buffer. After electrophoresis, the separated proteins were transferred to polyvinylidene difluoride membrane. After being blocked with 5% non-fat dry milk, the membrane with target proteins was incubated overnight at 4°C with antibodies against Eno-1, Mcee, Bdh1, Ces1c, Cbr4, ND2, Cox6a, Cox17, ATP 5j, Atl3, heat shock protein (HSP)70, HSP40, extracellular signal-regulated kinase (ERK) 1/2, P-ERK1/2, and CX 43 (Abcam, Cambridge, MA, United States). Blotted antibodies were visualized by HRP-conjugated second antibody (Cell Signaling Technology) and ECL detection system (Applygen Technologies, Beijing, China). Densitometric analyses of blots were performed using the Quantity One image analyzer software (Bio-Rad, Richmond CA, United States). The result of each band was expressed as relative optical density compared with internal controls of β-actin (Zheng et al., 2019).

### Statistical Analysis

All parameters were expressed as means ± SE. Statistical analysis was performed using one-way ANOVA, followed by Bonferroni test for multiple comparisons. A probability of less than 0.05 was considered statistically significant.

## Results

### T_89_ Improves ISO-Induced Rat Low Survival Rate and Restores MBF and Heart Function

**FIGURE 1:**
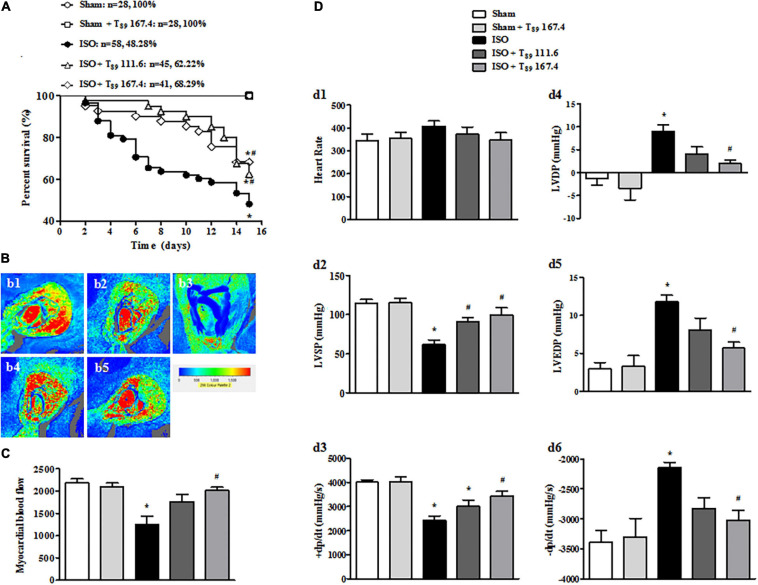
Effects of T_89_ on survival rate, MBF, and heart function in rats exposed to ISO. **(A)** The survival curve of each group. **(B)** Representative color images of MBF in different groups acquired by Laser-Doppler Perfusion Imager. b1: Sham; b2: Sham + T_89_ 167.4; b3: ISO; b4: ISO + T_89_ 111.6; b5: ISO + T_89_ 167.4. **(C)** Quantitative evaluation of MBF, *n* = 12. **(D)** Presented are the value of HR (d1), LVSP (d2), +dp/dt max (d3), LVDP (d4), LVEDP (d5), and -dp/dt max (d6) in various groups, *n* = 6. Data are presented as mean ± SE. *P < 0.05 vs. Sham group, ^#^*P* < 0.05 vs. ISO group.

As shown in Figure 1A, no rats in the Sham (*n* = 28) and Sham + T_89_ 167.4 group (*n* = 28) died during the whole experiment. The 15-day survival rate of rats in ISO group (*n* = 58) was 48.28%, significantly lower than that in the Sham group (^∗^*P* < 0.05 vs. Sham). However, compared with the ISO group, the survival rate of T_89_ treatment groups were significantly higher, which were 62.22% (*n* = 45 in the ISO + T_89_ 111.6 group) and 68.29% (*n* = 41 in the ISO + T_89_ 167.4 group), respectively (^#^*P* < 0.05 vs. ISO).

In view of improvement of rat survival, we wonder T_89_ could preserve the cardiac perfusion. Therefore, the effects of T_89_ on MBF were assessed. The representative images are shown in Figure 1B, which were manifested as distinct color with the red color representing the highest MBF and the blue color as the lowest. Impressively, MBF decreased after ISO in comparison with the Sham group (^∗^*P* < 0.05 vs. Sham), whereas treatment with T_89_ at dose of 167.4 mg/kg significantly attenuated ISO-induced decline in MBF (^#^*P* < 0.05 vs. ISO). The above results were confirmed by quantitative evaluation, as shown in Figure 1C.

The improvement of heart function contributes to elevation of rat survival rate, so heart function was detected in different groups. As demonstrated in Figure 1D, ISO administration caused a noticeable decline in LVSP (d2) and +dp/dt max (d3), and an inclement in LVDP (d4), LVEDP (d5), and -dp/dt max (d6) (^∗^*P* < 0.05 vs. Sham), indicating an impairment on heart function. However, this impairment was significantly alleviated by treatment with T_89_, especially at dose of 167.4 mg/kg (^#^*P* < 0.05 vs. ISO).

### T_89_ Retains Rat Myocardial Morphology and Ultrastructure and Alleviates Myocardial Injury Induced by ISO

**FIGURE 2:**
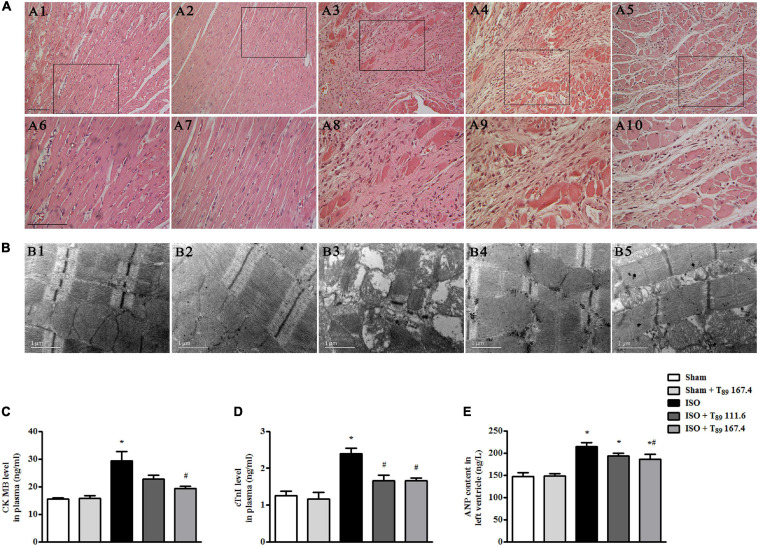
Effect of T_89_ on myocardial histology, ultrastructure, and myocardial injury after ISO. **(A)** Representative HE-stained images in Sham (A1, A6), Sham + T_89_ 167.4 group (A2, A7), ISO (A3, A8), ISO + T_89_ 111.6 (A4, A9), and ISO + T_89_ 167.4 (A5, A10). The upper panel was acquired at ×10 magnification of objective, and the lower panel was captured at ×20 magnification of objective. Bar = 100 μm. **(B)** Representative images of myocardial ultrastructure in Sham (B1), Sham + T_89_ 167.4 group (B2), ISO (B3), ISO + T_89_ 111.6 (B4), and ISO + T_89_ 167.4 (B5). **(C**–**E)** The alteration of CK MB (**C**, *n* = 6) and cTnI (**D**, *n* = 6) content in plasma, and ANP content (**E**, *n* = 12) in the left ventricle. *P < 0.05 vs. Sham group, ^#^*P* < 0.05 vs. ISO group.

Figure 2A displays the myocardial morphology stained by HE in Sham (A1 and A6), Sham + T_89_ 167.4 (A2 and A7), ISO (A3 and A8), ISO + T_89_ 111.6 (A4 and A9), and ISO + T_89_ 167.4 (A5 and A10) groups. The upper panels (A1 to A5) are representative micrographs captured under ×10 magnification of objective, and the lower panels (A6–A10) under ×20 magnification of objective. Compared with the Sham group, ISO elicited a distinct morphological injury, such as myocardial fiber disarrangement and disruption, leukocyte infiltration, and connective tissue hyperplasia. Obviously, T_89_ administration significantly preserved myocardium structure after ISO.

The myocardial ultrastructure in varies groups is presented in Figure 2B. In comparison with the Sham group (B1), ISO (B3) evoked significant alteration of myocardial ultrastructure, including myocardial fibers rupture, tissue edema, and mitochondrial swelling. Apparently, the ISO-induced alterations of myocardial ultrastructure were ameliorated in groups receiving T_89_ (B4 and B5).

To investigate the effect of T_89_ on myocardial injury, the level of CK MB and cTn I in plasma, and content of ANP in left ventricle were estimated by ELISA. Noticeably, the levels of CK MB (Figure 2C) and cTn I (Figure 2D) in plasma were significantly increased by ISO in comparison with the Sham group (^∗^*P* < 0.05 vs. Sham), while treatment with T_89_ remarkably inhibited ISO-induced elevation of CK MB and cTn I (^#^*P* < 0.05 vs. ISO). As shown in Figure 2E, ISO evoked obvious increase of ANP compared with the Sham group (^∗^*P* < 0.05 vs. Sham). While T_89_ 167.4 mg/kg treatment markedly reduced the elevation of ANP content caused by ISO (^#^*P* < 0.05 vs. ISO).

### Proteomics Analyses Reveal That the Regulation of T_89_ on ISO-Induced DEPs Mainly Enrich in Metabolic Pathways

**FIGURE 3:**
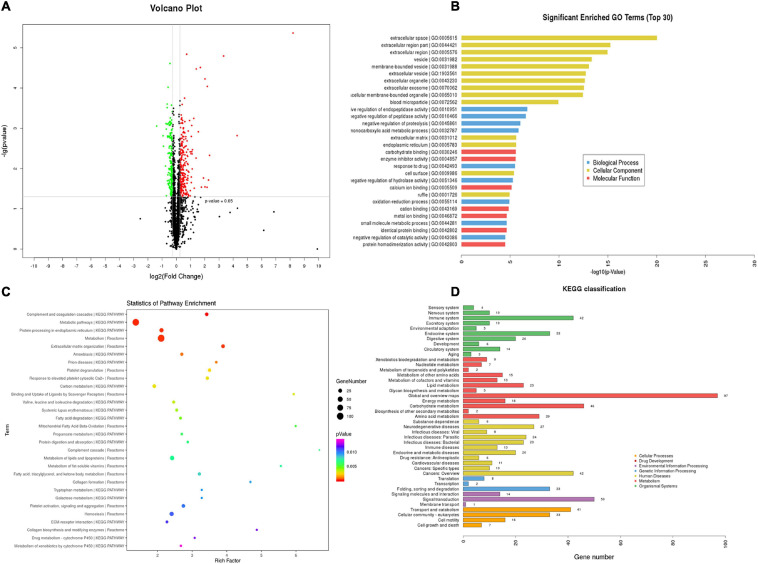
TMT-proteomics analysis of ISO group compared with the Sham group. **(A)** The Volcano Plot of differentially expressed proteins (DEPs) identified between ISO group and Sham group. Red dots represent upregulated DEPs; green dots represent downregulated DEPs; black dots represent unchanged proteins. **(B)** The top 30 significantly enriched GO terms for the DEPs identified between ISO group and Sham group using biological process, cellular component, and molecular function. **(C)** Statistics of pathway enrichment for the DEPs identified between ISO group and Sham group. **(D)** Classification of the enriched KEGG pathways for the DEPs identified between ISO group and Sham group.

TMT-quantitative proteomics were used to investigate the protective mechanisms of T_89_. A total of 4,092 proteins and 30,319 peptides were successfully identified by LC-MS/MS, and 1201 DEPs were detected among the three groups. A total of 516 DEPs were identified between the ISO group and Sham group, 293 of which were upregulated, while 223 were downregulated. The Volcano Plot of ISO vs. Sham is shown in Figure 3A. The top 30 GO terms between the ISO and Sham group that showed remarkable enrichment are shown in Figure 3B. Biological process (BP) analysis showed that the majority of the DEPs identified between ISO and Sham were classified as “negative regulation of enzyme activity” and “biological metabolic process.” With regard to cellular component (CC) terms, most of the DEPs identified between the ISO and Sham groups were associated with the extracellular and membrane-bounded vesicles. With regard to molecular function (MF), the identified DEPs were predominantly involved in binding processes, and in particular, ionic binding and protein binding. Next, the biological pathway enrichment of DEPs identified between ISO and Sham groups were statistically analyzed. As shown in Figure 3C, either KEGG pathway or Reactome annotation displayed that the main signaling pathways undergoing modulation were those related to metabolism. Furthermore, KEGG pathway detail analysis was performed and showed in Figure 3D, demonstrating 46, 23, and 16 DEPs being related to carbohydrate metabolism, lipid metabolism and energy metabolism, respectively.

**FIGURE 4:**
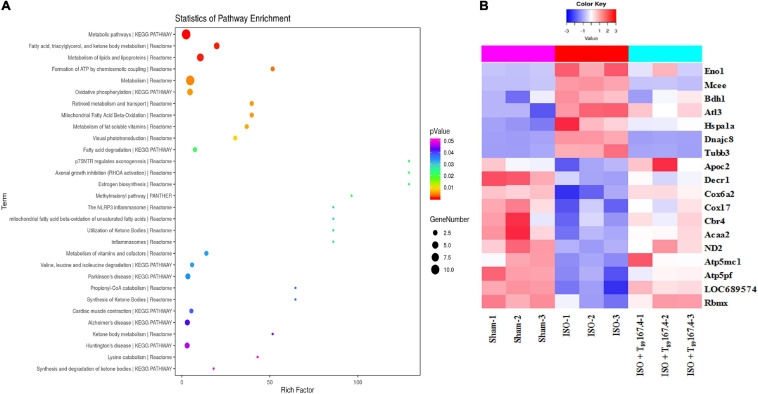
TMT-proteomics analysis of ISO + T_89_ group compared with the ISO group. **(A)** Statistics of pathway enrichment for the DEPs identified between ISO + T_89_ group and ISO group. **(B)** The heat map of DEPs altered in Sham, ISO and ISO + T_89_ group.

Statistical analysis of pathway enrichment between ISO + T_89_ 167.4 group and ISO group disclosed that the greatest changes occurred in fatty acid metabolism and ATP formation pathway (Figure 4A). The heat maps of DEP variations among Sham, ISO, and ISO + T_89_ 167.4 groups are displayed in Figure 4B, including Eno1, Mcee, Bdh1, Apoc2, Decr1, Cbr4, Acaa2, ND2, Cox6a, Cox17, ATP5mc1, ATP5pf, etc.

### T_89_ Inhibits Glycolysis and Promotes Fatty Acid Metabolism in Rats Exposed to ISO by Regulating Glycolipid Metabolism-Related Enzymes

**FIGURE 5:**
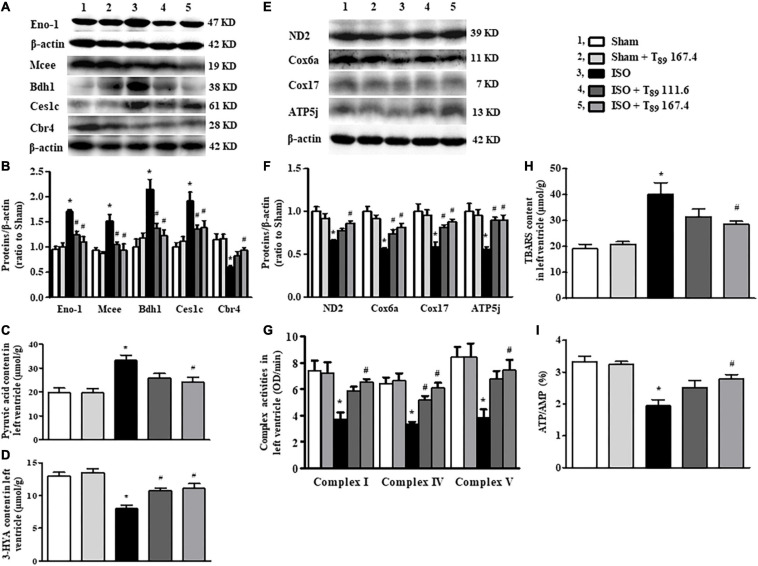
Effect of T_89_ on ISO-induced alteration in metabolism and mitochondrial function. **(A**,**B)** Representative Western blotting bands and quantitative analysis of Eno1, Mcee, Bdh1, Ces1c, and Cbr4 in various groups. *n* = 4. **(C**,**D)** The content of pyruvic acid **(C)** and 3-HYA **(D)** in left ventricles in different groups. *n* = 6. **(E**,**F)** Representative Western blotting bands and quantitative analysis of ND2, Cox6a, Cox17, and ATP5j in various groups. *n* = 4. **(G)** The activity of complex I, IV, and V in left ventricles. *n* = 6. **(H**,**I)** The level of TBARS **(H)**, and the ratio of ATP/AMP **(I)** in left ventricles. *n* = 6. *P < 0.05 vs. Sham group, ^#^*P* < 0.05 vs. ISO group.

Carbohydrate metabolism alteration was found as the main metabolic pathway changed after ISO challenge. As shown in Table 1, in comparison with the Sham group, ISO reduced 31 proteins and increased 15 proteins that are associated with carbohydrate metabolism. The 31 reduced proteins were mainly involved in citrate cycle, gluconeogenesis, and other pathways that inhibit glucose synthesis. Whereas, the 15 increased proteins were mainly enriched in glycolysis, indicating enhancement of glucose oxygenolysis. These alterations of carbohydrate metabolism implied that more carbohydrates were mobilized to provide energy through glycolysis after ISO challenge. Of the above changes, T_89_ prominently inhibited the expression of Eno1, which was confirmed by Western blot (Figure 5A).

**TABLE 1:** The altered proteins enriched in carbohydrate metabolism pathways evoked by ISO.

Carbohydrate metabolism pathways	Altered proteins (ISO vs. Sham)	Up or down
Glycolysis	Eno1, Eno2, Eno3, Pfkp, Hk1, Hk2	Up
Pentose phosphate pathway	G6pd, Tkt, Pfkp	Up
Citrate cycle	Mdh1, Suclg1, Suclg2, Idh1, Idh2	Down
Glyoxylate and dicarboxylate metabolism	Cat, Mut, Mdh1, Pgp, Pcca, Acat1	Down
Pentose and glucuronate interconversions	Akr1b10, Akr1b1, Sord, Dcxr	Down
Pyruvate metabolism	Mdh1, Aldh9a1, Glo1, Acyp2, Ldhd, Acat1	Down
Galactose metabolism	Gaa, Galt, Akr1b1, Akr1b10	Down
Glycerolipid metabolism	Akr1b10, Aldh9a1, Akr1b1	Down
Fructose and mannose metabolism	Akr1b10, Akr1b1, Sord	Down
Gluconeogenesis	Aldh9a1, Adh5	Down
Starch and sucrose metabolism	Gaa, Pygb	Down

*ISO-induced altered proteins enriched in carbohydrate metabolism pathways. Acat1, acetyl-CoA acetyltransferase; Acyp2, acylphosphatase-2; Adh5, alcohol dehydrogenase class-3; Aldh9a1, 4-trimethylaminobutyraldehyde dehydrogenase; Akr1b1, aldose reductase; Akr1b10, aldo-keto reductase family 1 member B10; Cat, catalase; Mut, methylmalonyl-CoA mutase; Dcxr, L-xylulose reductase; Eno1, alpha-enolase; Eno2, gamma-enolase; Eno3, beta-enolase; G6pd, glucose-6-phosphate 1-dehydrogenase; Gaa, lysosomal alpha-glucosidase precursor; Galt, galactose-1-phosphate uridylyltransferase; Glo1, lactoylglutathione lyase; Hk1, hexokinase-1; Hk2, hexokinase-2; Idh1, isocitrate dehydrogenase [NADP] cytoplasmic; Idh2, isocitrate dehydrogenase [NADP]; Ldhd, probable D-lactate dehydrogenase, mitochondrial isoform X1; Mdh1, malate dehydrogenase; Pcca, propionyl-CoA carboxylase alpha chain; Pfkp, ATP-dependent 6-phosphofructokinase; Pgp, glycerol-3-phosphate phosphatase; Pygb, glycogen phosphorylase; Sord, sorbitol dehydrogenase; Suclg1, succinate–CoA ligase [ADP/GDP-forming] subunit alpha; Suclg2, succinyl-CoA ligase [GDP-forming] subunit beta; Tkt, transketolase isoform X1.*

Carbohydrate metabolism alteration is closely linked to lipid metabolism. As shown in Table 2, ISO challenge elicited upregulation of seven lipid metabolism proteins and downregulation of 16 proteins. Among them, the enhancement of Mcee, Bdh1, and Ces1c were apparently reversed by T_89_, which was confirmed by Western blotting analysis (Figures 5A,B). In contrast, the 16 reduced proteins by ISO were mainly enriched in lipid digestion, mobilization and transport, and fatty acid β-oxidation. The remarkable decline of these proteins suggested an inclement of fatty acid metabolism after ISO. The above inclement was partly alleviated by T_89_, manifesting as the significant enhancement of Apoc2, Decr1, Acaa2, and Cbr4 (Figure 4B).

**TABLE 2:** The altered proteins enriched in lipid metabolism pathways evoked by ISO.

Lipid metabolism pathways	Altered proteins (ISO vs. Sham)	Up or down
Triglycerides metabolism	Agpat3, Ppt1, Lclat1, Ces1c	Up
Arachidonic acid metabolism	Ptgis	Up
Short-chain fatty acid catabolism	Mcee	Up
Synthesis and degradation of ketone bodies	Bdh1	Up
Long chain omega-hydroxy fatty acids oxidation	Adh4	Down
Lipid digestion, mobilization and transport	Fabp3, Apoc2, Lp1, Gpcpd1	Down
Fatty acyl-CoA biosynthesis	Acsf2, Acsl1, Tecr, Cbr4, Gcdh	Down
Mitochondrial fatty acid β-oxidation	Acaa2, Acat1, Decr1, Eci1, Echs1, Gcdh, Hadh, Hadhb	Down

*ISO-induced altered proteins enriched in lipid metabolism pathways. Acaa2, 3-ketoacyl-CoA thiolase; Acat1, acetyl-CoA acetyltransferase; Acsf2, acyl-CoA synthetase family member 2; Acsl1, long-chain-fatty-acid–CoA ligase 1; Adh5, alcohol dehydrogenase class-3; Agpat3, 1-acyl-sn-glycerol-3-phosphate acyltransferase gamma; Apoc2, apolipoprotein C-II precursor; Bdh1, D-beta-hydroxybutyrate dehydrogenase; Cbr4, carbonyl reductase family member 4; Ces1c, carboxylesterase 1C; Decr1, 2,4-dienoyl-CoA reductase; Echs1, enoyl-CoA hydratase; Eci1, enoyl-CoA delta isomerase 1; Fabp3, fatty acid-binding protein; Gcdh, glutaryl-CoA dehydrogenase; Gpcpd1, glycerophosphocholine phosphodiesterase; Hadh, trifunctional enzyme subunit beta; Hadhb, trifunctional enzyme subunit beta; Lclat1, lysocardiolipin acyltransferase 1; Lp1, lipoprotein lipase precursor; Mcee, methylmalonyl-CoA epimerase; Ppt1, palmitoyl-protein thioesterase 1 precursor; Ptgis, prostacyclin synthase precursor; Tecr, very-long-chain enoyl-CoA reductase.*

Consistent with enhancement of glycolysis and inclement of fatty acid oxidation after ISO, ISO evoked an obvious increase of pyruvic acid (Figure 5C), whereas a remarkable decline of 3-HYA (Figure 5D) in the left ventricle (^∗^*P* < 0.05 vs. Sham). The above alterations induced by ISO were significantly alleviated by T_89_ (^#^*P* < 0.05 vs. ISO).

### T_89_ Attenuates ISO-Induced Energy Metabolism Disorders and Oxidative Stress Injury by Improving ND2, Cox6a, Cox17, ATP5g, and ATP 5j Expression and Restoring Mitochondrial ETC Complex Activities

The alterations in glycolipid metabolism inevitably lead to energy metabolism disorders, which mainly manifests as oxidative phosphorylation dysfunction. Not surprisingly, ISO reduced 16 energy metabolism proteins, of which 13 proteins (ND1, ND2, Ndufa5, Ndufa10, Ndufa11, Ndufab1, Cox5a, Cox6b1, Cox6a2, Cox17, ATP5fle, ATP5g, and ATP5j) were subunits of mitochondrial ETC complexes, engaged in oxidative phosphorylation. Excitingly, five subunits reduced by ISO were significantly reversed by T_89_, they were ND2 (subunit of complex I), Cox6a, and Cox17 (subunits of complex IV), ATP 5g, and ATP 5j (subunits of complex V), which was confirmed by Western blotting analysis (Figures 5E,F). Accordingly, the activities of complex I, IV, and V were remarkably inhibited after ISO (^∗^*P* < 0.05 vs. Sham) and were obviously restored by T_89_ (^#^*P* < 0.05 vs. ISO) (Figure 5G). These data highlight the involvement of mitochondrial function in the beneficial role of T_89_.

To further evaluate the effect of mitochondrial function on oxidative stress and energy metabolite, the contents of TBARS, AMP, and ATP in the left ventricle were determined by ELISA. As shown in Figures 5H,I, ISO elicited an obvious elevation of TBARS and a significant decline of ATP/AMP compared with the Sham group (^∗^*P* < 0.05 vs. Sham). On the contrary, T_89_ at 167.4 mg/kg significantly reversed the alteration of TBARS and ATP/AMP caused by ISO (^#^*P* < 0.05 vs. ISO).

### The Cardioprotective Effect of T_89_ Implicates the Regulation of HSP70, HSP40, P-ERK, and CX43 After ISO

**FIGURE 6:**
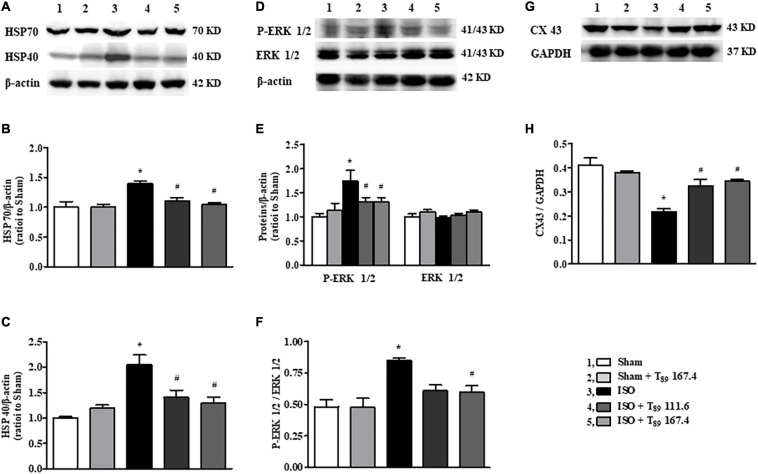
Effect of T_89_ on ISO-induced alteration in HSP70, HSP40, ERK 1/2, and CX43. **(A–C)** Western blotting validation of HSP70, HSP40, and corresponding quantitative evaluation in different groups. *n* = 4. **(D**–**F)** Representative Western blotting bands of P-ERK1/2, ERK 1/2, and the quantitative evaluation in different groups. *n* = 4. **(G**,**H)** Representative Western blotting bands and the quantitative analysis of CX43 in different groups. *n* = 4. *P < 0.05 vs. Sham group, ^#^*P* < 0.05 vs. ISO group.

In addition to metabolism-related proteins, proteomics analysis showed that T_89_ could also regulate other proteins, such as Hspa1a (HSP70), and Dnajc8 (HSP40). Western blotting examination (Figures 6A–H) demonstrated that ISO-induced elevation of HSP70, HSP40 and p-ERK 1/2 and reduction of CX43, indicating that these proteins are involved in cardiac hypertrophy induced by ISO. Nevertheless, all the alteration caused by ISO was significantly reversed by T_89_ treatment (^#^*P* < 0.05 vs. ISO).

### T_89_ Alleviates ISO-Induced Rat Cardiac Hypertrophy

**FIGURE 7:**
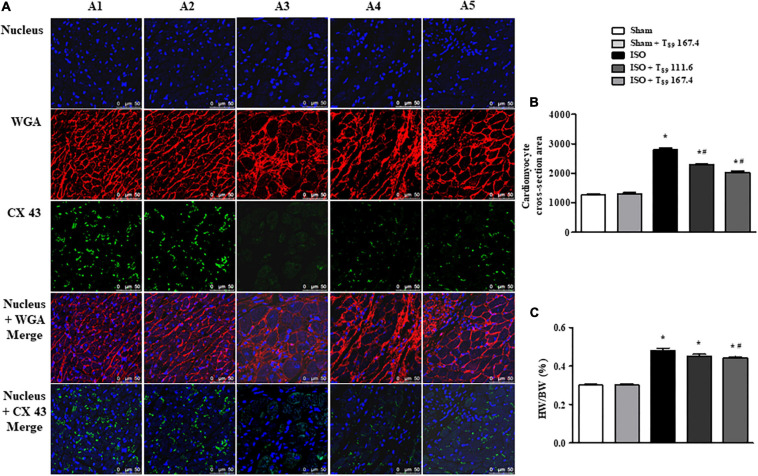
Effect of T_89_ on ISO-induced cardiac hypertrophy. **(A)** Representative images of immunofluorescent staining of WGA and CX 43 in Sham (A1), Sham + T_89_ 167.4 group (A2), ISO (A3), ISO + T_89_ 111.6 (A4), and ISO + T_89_ 167.4 (A5) groups. WGA is stained red, CX 43 is stained green, and nucleus blue. Bar = 50 μm. **(B)** Quantitative analysis of cardiomyocyte cross-section area. *n* = 4. **(C)** The ratio of HW/BW in each group. *n* = 11. *P < 0.05 vs. Sham group, ^#^*P* < 0.05 vs. ISO group.

Wheat germ agglutinin is used to demarcate cell boundaries for determination of cardiomyocyte size. CX 43 is immunolabeled in the interstitial disk as markers of the gap junction of cardiomyocytes. Figure 7A shows the images of WGA and CX 43 by immunofluorescent staining in each group, where WGA is red and CX 43 is green. Obviously, in the Sham (A1) and Sham + T_89_ 167.4 (A2) groups, the myocardial boundaries stained by WGA were complete and distinct, and CX 43 expression was abundant. Compared with the Sham group, the cardiomyocyte boundary was notably enlarged and CX 43 expression was significantly decreased in ISO group (A3). However, T_89_ treatment (A4 and A5) inhibited the enlargement of cardiomyocyte boundary and the breakdown of CX 43 caused by ISO.

The quantitative analysis of cardiomyocyte cross-sectional area is presented in Figure 7B, revealing that ISO elicited a remarkable augment on cardiomyocyte cross-sectional area, which was alleviated by T_89_ (^∗^*P* < 0.05 vs. Sham, ^#^*P* < 0.05 vs. ISO). To evaluate the hypertrophic response to ISO, the ratio of HW/BW was measured. As noticed in Figure 7C, ISO educed obvious increase of HW/BW compared with the Sham group (^∗^*P* < 0.05 vs. Sham), whereas T_89_, especially at dose of 167.4 mg/kg, significantly inhibited ISO-induced elevation of HW/BW (^#^*P* < 0.05 vs. ISO).

## Discussion

In the present study, we first confirmed that treatment with T_89_ notably elevated ISO-induced low survival rate of rats and improved cardiac injury, mainly manifesting as alleviation of cardiac hypertrophy, preservation of myocardial morphology, and ultrastructure, thus resulting in restoration of MBF and heart function. Quantitative proteomics analysis demonstrated T_89_ improved metabolic disorders by regulating expression of Eno1, Mcee, Bdh1, Ces1c, Apoc2, Decr1, Acaa2, Cbr4, ND2, Cox6a, Cox17, ATP5g, and ATP5j, further restoring mitochondrial ETC complex activities and attenuating ISO-caused oxidative stress damage and energy depletion. The regulation of metabolism and recovery of mitochondrial function by T_89_ may be the underlying mechanism contributing to the potential effect of T_89_ on ISO-elicited cardiac injury. Furthermore, study on signaling verified that the cardioprotective effect of T_89_ implicated HSP70, HSP40, and ERK 1/2 signals activation.

Isoproterenol-induced cardiac injury model used in the present study has some similarity in pathogenesis to that of myocardial ischemia without significant coronary artery obstruction in that the condition implicates metabolic disorders (Mukherjee et al., 2012). Disorders in metabolism will lead to low rat survival rate due to imbalance of oxygen supply and demand. Unsurprisingly, we observed a lower level of MBF in rats exposed to ISO, suggesting the shortage of oxygen and nutrition, which is responsible for changes in the energy metabolism substrates by cardiomyocytes, and hence resulting in a metabolic shift from primarily fatty acid metabolism to glycolysis (Quan et al., 2017; Szablewski, 2017). Nevertheless, we observed T_89_ obviously reduced mortality and improved outcome in ISO-induced rat cardiac injury model.

Previous studies by plasma and urine metabolism analysis showed that compound Danshen dripping pills improve myocardial ischemia by regulating glycolipid metabolism (Xin et al., 2013; Zou et al., 2015). The results in the present study provided support for these results showing that T_89_ reversed glycolysis elevation and fatty acid metabolism inhibition in response to ISO chronic stimuli and further demonstrated that the effect of T_89_ on regulating glycolipid metabolism depended on regulation of related proteins. As confirmed by Western blotting examination, T_89_ significantly reversed the expression pattern of glycolytic enzyme Eno-1 caused by ISO, similar to that in models of pressure overload-induced cardiac hypertrophy (Chen et al., 2015). Moreover, T_89_ could regulate the increase of Mcee, Bdh1, and Ces1c and decrease of Apoc2, Decr1, Acaa2, and Cbr4 induced by ISO, which were associated with lipid metabolism. Additionally, the DEPs could influence other biological activity of T_89_ in different ways. For example, Mcee gene provides instructions for making an enzyme called methylmalonyl CoA epimerase, which converts one form of the molecule methylmalonyl CoA to another, while Ces1c is responsible for the hydrolysis of ester- and amide-bond-containing xenobiotics and drugs (Wong et al., 2015; Pan et al., 2017). Acaa2 catalyzes the last step of the mitochondrial fatty acid β-oxidation spiral and has been shown to be a functional BCL2-interacting protein 3 binding partner (Cao et al., 2008), which provides a possible link between fatty acid metabolism and cell apoptosis. In response to the modulation of glycolipid metabolism-related enzymes, ISO-induced pyruvic acid and 3-HYA content in left ventricle was recovery by T_89_, implying that T_89_ altered the ISO-evoked metabolic state, making fatty acid metabolism a predominant energy provider.

Glycolipid metabolism imbalance is responsible for energy metabolism disorder, which is closely related to mitochondrial dysfunction (Zhou and Tian, 2018). On the one hand, the elevation of glycolysis and inhibition of fatty acid metabolism in response to ISO will lead to reduction of acetyl coenzyme, as well as the reduction of NADH and FADH2, which are the raw materials for oxidative phosphorylation to produce ATP in mitochondrion (Wilson, 2017; Davila et al., 2018). On the other hand, impaired mitochondria not only fail to produce enough ATP but also become a major source of reactive oxygen species (ROS) due to the leakage of electrons from disrupted ETC (Peoples et al., 2019). In the present study, we observed ISO evoked a decrease in the activity of mitochondrion complex I (NADH dehydrogenase complex), complex IV (cytochrome *c* oxidase), and complex V (ATP synthase), which lead to energy depletion and oxidative stress injury. Proteomics analysis revealed that the reduction of complexes activities depended on the downregulation of their subunits. However, T_89_ treatment apparently reversed ISO-caused partial subunits reduction, including complex I subunit ND2, complex IV subunits Cox6a and Cox17, and complex V subunits ATP5g and ATP5j. These effects at least partly contributed to the restoration of Complexes activities by T_89_.

Energy metabolism disorder is well recognized to account for cardiac injury *via* necrosis and/or apoptosis (Willis et al., 2015). As expected, ISO resulted in a destructed morphology of myocardial tissue. T_89_ protected myocardial injury after ISO stimulation, most likely *via* preservation of mitochondria. This is verified by the alteration of ultrastructure, showing that T_89_ alleviated ISO-induced mitochondrial swelling and vacuolization.

The improvement effect of T_89_ on energy metabolism may also be related to its regulation of signal molecules, such as Hspa1a and Dnajc8, which encodes HSP70 and HSP40, respectively. HSP70 is implicated in a wide variety of cellular processes, implementing its function through ATPase cycle mediated by its co-chaperones such as HSP40. HSP40 was revealed to stimulate ATPase hydrolysis by HSP70, when HSP70 underwent a conformational change it increased its affinity for substrate proteins (Jakobsson et al., 2013). It has been reported that HSP70 could bind to Eno1, the latter was required not only for glycolysis but also for hypoxia tolerance and ATP biosynthesis process (Luo et al., 2011). In line with the above reports, the present results suggested that ISO challenge stimulated ATP hydrolysis to ADP by increasing HSP70 and HSP40 expression, leading to low affinity of HSP70 with Eno1, thus inhibiting the biosynthesis process of ATP. However, T_89_ administration could block the above process by downregulating the expression of HSP70, HSP40, and Eno1.

HSP70 was reported to promote cardiac hypertrophy development *via* activating prohypertrophy signal ERK 1/2 (Cai et al., 2010; Somensi et al., 2017). Consistent with these reports, the present study revealed that ISO chronic stimuli apparently enhanced the phosphorylation of ERK 1/2, leading to a remarkable augment on cardiomyocyte cross-sectional area and the ratio of HW/BW. Meanwhile, myocardial CX43 expression was significantly decreased and redistributed following cardiac hypertrophy. Noticeably, T_89_ inhibited the activation of ERK 1/2 and the redistribution of CX43, finally attenuating cardiac hypertrophy. The alleviation of cardiac hypertrophy might have contributed to the potential preservation of heart function and elevation of rat survival rate.

In addition to regulating metabolic pathway, the DEPs identified in the present study may affect other biological activity of T_89_. For example, Atl3, one of atlastins, is a class of membrane-bound dynamin-like GTPase that is commonly believed to promote tubular endoplasmic reticulum (ER) fusion, and also function as an autophagy receptor to mediate ER tubule degradation and leading to ER-phagy dysfunction (Chen et al., 2019). The present study revealed that Atl3 was upregulated by ISO stimuli, implying that ISO may cause autophagy and ER-phagy dysfunction, while these alterations could be reversed by T_89_. However, the involvement of autophagy and ER-phagy dysfunction in ISO-induced model and the effect of CP on it need further research.

## Conclusion

**FIGURE 8:**
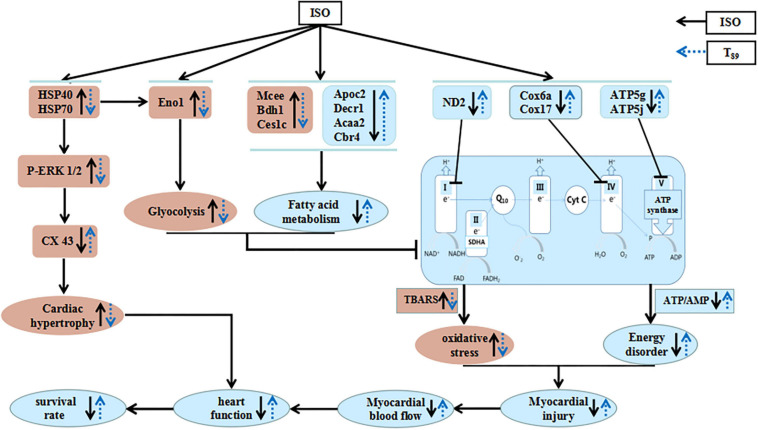
A diagrammatic sketch showing the pathways that lead to the protected effects of T_89_ on ISO-induced cardiac injury. ISO causes enhancement of glycolysis and reduction of fatty acid metabolism and oxidative phosphorylation, resulting in energy disorder and oxidative stress with myocardial injury and reduction of MBF. Meanwhile, ISO-educed cardiac hypertrophy *via* regulating HSP70-ERK1/2 signals, which, together with cardiac injury, finally caused reduction of heart function and low survival rate. Nevertheless, T_89_ administration reversed all the above alteration. The cardioprotective role of T_89_ depended on the regulation of glycolipid metabolism, recovery of mitochondrial function, and improvement of myocardial energy.

As summarized in Figure 8, the present study demonstrated the potential of T_89_ to protect ISO-induced myocardial injury, as evidenced by restoration of MBF and heart function, preservation of myocardial morphology, and attenuation of cardiac hypertrophy with elevating survival rates. The cardioprotective role of T_89_ is correlated with the regulation of glycolipid metabolism, recovery of mitochondrial function, and improvement of myocardial energy, implicating modulation on Eno1, Mcee, Bdh1, Ces1c, Apoc2, Decr1, Acaa2, Cbr4, ND2, Cox6a, Cox17, ATP5g, ATP5j, HSP70, HSP40, p-ERK 1/2, and CX43 molecules.

## Data Availability Statement

The datasets presented in this study can be found in online repositories. The names of the repository/repositories and accession number(s) can be found below: Proteomics data are available via 59 ProteomeXchange with identifier PXD024641.

## Ethics Statement

The animal study was reviewed and approved by the Peking University Biomedical Ethics Committee Experimental Animal Ethics Branch.

## Author Contributions

J-YH was designer of the whole thesis. X-HW participated in all experiments. XG, C-SP, HL, Y-CC, S-YH, J-ND, L-LY, KS, and C-SW contributed to experiment of animal model and reagent preparation. LY, B-HH, and XC contributed to ultrastructure examination. J-YF provided amendment opinions for the study. All authors contributed to the article and approved the submitted version.

## Conflict of Interest

The authors declare that the research was conducted in the absence of any commercial or financial relationships that could be construed as a potential conflict of interest.
